# Computerized Cognitive Training by Healthy Older and Younger Adults: Age Comparisons of Overall Efficacy and Selective Effects on Cognition

**DOI:** 10.3389/fneur.2020.564317

**Published:** 2021-01-08

**Authors:** Nicole F. Ng, Allen M. Osman, Kelsey R. Kerlan, P. Murali Doraiswamy, Robert J. Schafer

**Affiliations:** ^1^Department of Research and Development, Lumos Labs, San Francisco, CA, United States; ^2^Duke University School of Medicine, Neurocognitive Disorders Program, Department of Psychiatry and Behavioral Sciences, Duke Institute for Brain Sciences, Durham, NC, United States

**Keywords:** computerized cognitive training, cognitive aging, age-related cognitive decline, mild cognitive impairment, Alzheimer's disease, digital health, telemedicine

## Abstract

Among the non-pharmacological methods under development for maintaining cognitive function across the lifespan is computerized cognitive training (CCT). There has been considerable interest in using CCT to slow or remediate age-related cognitive decline, both normal and pathological. Toward these ends, it would be useful to know how the effects of CCT on cognitive function vary over the course of normal cognitive aging. Are there changes in either 1) the overall efficacy of CCT or 2) which cognitive faculties are affected? To address these two questions, we reanalyzed results from a large online study by Hardy et al. (1) of 4,715 adults between 18 and 80 that examined effects of CCT on both a neuropsychological test battery and self-reported ratings of cognition and affect in daily living. Combined across all participants, Hardy et al. found greater improvement on both types of assessment following 10 weeks of CCT with the commercial program Lumosity, as compared to practice with a control activity involving computerized crossword puzzles. The present study compared the size of these effects on the older (50–80) and younger (18–49) participants. To address the question of overall efficacy, we examined CCT effects (treatment minus control) on overall performance of the test battery and mean rating. No significant difference on either measure was found between the two age cohorts. To address the question of whether the same magnitude of overall effects on both age cohorts was due to equivalent effects on the same set of underlying cognitive functions, we examined the patterns of CCT effects across individual subtests and rated items. These patterns did not differ significantly between the two age cohorts. Our findings suggest that benefits from CCT can occur to a similar degree and in a similar way across an extended part of the adult lifespan. Moreover, the overall effects of CCT delivered over the internet were of the same small to medium size as those typically found in the lab or clinic. Besides improving access and reducing the cost of CCT for older adults, delivery over the internet makes long-term training more practicable, which could potentially yield larger benefits.

## Introduction

As the global population ages (2) and the worldwide incidence of Alzheimer's and other dementias continues to rise (3), maintenance of cognitive function across the lifespan has become increasingly important (4–6). Some declines in cognitive function are considered to be normal consequences of aging. The current literature on typical cognitive aging (Age-Related Cognitive Decline, ARCD) highlights a gradual decline in key cognitive domains including memory, learning, language, and processing speed (7–9). Dementias involve a decline in memory and other cognitive functions severe enough to interfere with activities of daily living (ADLs) (10, 11). While there are different types of dementia with different etiologies and symptoms, all are distinguished from ARCD by their negative impact on ADLs, which results in a loss of independence. The relations between ARCD and dementia are unclear (5). There exists however an intermediate level of cognitive decline, termed Mild Cognitive Impairment (MCI), that is a risk factor for subsequent development of dementia (12). Diagnosis of MCI is based on first- and/or third-person reports of cognitive decline and performance below age norms on neuropsychological tests, without a diagnosis of dementia and with preserved ability to perform ADLs (13). MCI is thought to arise from early, prodromal stages of disease processes causing dementia (13, 14).

Among the approaches for maintaining cognitive function are behavioral ones intended to stimulate the brain. That engagement in cognitively stimulating activities throughout life can have positive effects on late-life cognition is suggested by many observational studies (4). Among the factors used to estimate the amount of cognitive engagement have been years of education, type of occupation, and amount of participation in cognitively stimulating leisure activities (15). Associations have been found between such factors and the degree of normal ARCD [e.g., (16)], incidence of MCI [e.g., (17)], and incidence of dementia [e.g., (18)]. Moreover, while precise mechanisms remain to be specified, there exist plausible scenarios involving neural plasticity by which effects of cognitive engagement on cognitive aging might occur. Cognitive engagement might enhance or preserve declining brain structures or functions [e.g., (19)], or it might recruit additional circuitry that provides compensation (20, 21). The possibility of reducing cognitive decline or its impact through cognitive engagement has led to the targeted examination of both traditional activities and novel interventions.

The present study concerns one such intervention: computerized cognitive training (CCT). The effects of CCT on older adults have been studied experimentally in randomized controlled trials (RCTs). These, of necessity, have involved only limited periods of training, followed sometimes by booster sessions. Nonetheless, a number of large methodologically sophisticated RCTs have reported benefits of CCT on ARCD (4–6). Examples include the Advanced Cognitive Training for Independent and Vital Elderly study [ACTIVE; (22)], the Iowa Healthy and Active Minds Study [IHAMS; (23, 24)], and a study performed by Corbett et al. (25). Each of these studies found positive effects of CCT on both neuropsychological tests of cognition and self-reported ratings of ADLs. Moreover, in the ACTIVE study, both types of benefit were still observed five (26) and ten (27) years after training. The Corbett et al. study is noteworthy in that it showed that CCT administered over the internet and involving programs similar to commercially available ones can have beneficial effects on older adults. Benefits of CCT similar to those found for healthy older adults have been found also in cohorts with MCI (28). In contrast, an impact on the incidence or onset of MCI or dementia has still to be demonstrated convincingly (5, 6). But such evidence may be forthcoming from ongoing or future RCTs [e.g., (29)].

To better assess and understand the impact of CCT on older adults, it would be useful to know how this impact compares with that on younger adults receiving the same training. Are there differences in either the overall efficacy of CCT or which cognitive faculties are affected? Given the decreased neural plasticity of older adults, one might expect less of a neural effect of any cognitive treatment. On the other hand, given their diminished neural resources, the same neural effects might translate into larger cognitive or behavioral effects for older adults. Besides quantitative differences, there might also be qualitative ones between the effects of CCT on old vs. young adults. Such differences might involve the identities of affected cognitive faculties or relative sizes of effects on the same set of faculties. Understanding how the effects of CCT are modulated by normal cognitive aging could provide clues as to whether CCT strengthens or slows decline of faculties that typically decline with age, as opposed to strengthening faculties that compensate for declining ones. It would also seem to be a natural step toward understanding and maximizing the impact of CCT on late pathological stages.

We therefore sought to provide a direct comparison of the cognitive effects of CCT on old vs. young adults. Such comparisons have been reported in a number of studies so far. But these studies have been small, and thus lack the power to test hypotheses concerning effects of the size typically found in larger studies and meta-analyses of CCT. Integrating results across these studies is difficult because they are heterogeneous in a number of ways, including targeted cognitive faculties, CCT tasks, control-group tasks, and outcome measures. While these challenges can be overcome to some extent in meta-analyses [e.g., (30); but see (31)], it would be highly informative to compare the effects of CCT on old vs. young participants within the same large study. The online study of CCT by Hardy et al. (1), with analyzable results from 4,715 healthy participants aged 18 to 80, provided such an opportunity.

Hardy et al. (1) performed a randomized control trial of CCT in which the treatment group trained with the commercial online application Lumosity. As in the IHAMS study, the active control group solved crossword puzzles. The outcome measures involved both a clinical neuropsychological test battery and a survey assessing cognition and affect during daily living. The treatment group improved significantly more than the control group on aggregate measures of the test battery and survey. Participants in the treatment group also showed significantly greater improvements on specific subtests of the test battery, including speed of processing, working memory, problem solving, and fluid intelligence. They likewise showed significantly greater improvement on individual survey items, especially those related to concentration. While the study found positive effects of CCT on adults in general, it did not compare effects on older vs. younger adults.

Here we compare the efficacy of CCT in enhancing both cognitive performance and self-reported real world experience for older vs. younger adults in the Hardy et al. (1) study. As in the IHAMS and Corbett et al. (25) studies, the older cohort consisted of participants 50+ years of age. The primary goal was to compare older and younger adults with respect to the size of the overall effect of CCT on the performance and survey measures. A secondary goal concerned possible qualitative differences between the effects of CCT on the two age cohorts. Qualitative differences were assessed by comparing the two age cohorts on their profiles of CCT effects across the individual neuropsychological subtests and across the individual survey items.

## Methods

Results from the Hardy et al. (1) study were reanalyzed in order to compare the effects of cognitive training on older and younger adults.

### Ethical Statement

Hardy et al. [(1); clinicaltrials.gov identifier NCT02367898] was reviewed and approved by Ethical and Independent Review Services (IRB Protocol 13054-01). Written informed consent was obtained from all participants prior to their study enrollment. Review, approval, and informed consent were not required for the subsequent analyses reported here.

### Design and Procedure

A detailed description of the study is presented in Hardy et al. (1). In brief, potential participants were selected from among a subset of Lumosity users who registered for the free and limited version of the program. Participants were invited via email to take part in a cognitive training study. After providing informed consent, participants were given a computerized neuropsychological test battery, the NeuroCognitive Performance Test [NCPT; (32)] and an online survey of real-world cognitive performance. Participants were then randomized into either the cognitive training condition, which consisted of full access to Lumosity, or into an active control condition that consisted of daily crossword puzzles delivered online. The cognitive training and control conditions are described in Hardy et al. Participants were instructed to train for 10 weeks, and at the end of this period were invited to take the NCPT and the survey a second time.

### Participants

Participation in the Hardy et al. (1) study was limited to individuals between 18 and 80 years of age. The present study divided these participants into two age cohorts. Those 50 years or older were included in the older adult cohort and those younger than 50 were included in the younger adult cohort. The age cutoff of 50 corresponds to the minimum for inclusion in the IHAMS and Corbett et al. (25) studies and provided a sufficient number of participants in both cohorts. It also seemed reasonable given that the IHAMS study found no differences in the size of cognitive training effects between those 50–64 and 65+. Moreover, prior research involving the NCPT (32) indicated that there should be a considerable difference in baseline performance between the two age cohorts.

A consort flow chart involving the 4,715 participants examined in the present study can be found in Hardy et al. (1). Information about each separate age cohort is presented in the following summary. Of the individuals who took the NCPT pre-test, 10,198 were between the ages of 18 and 80 (2,185 older & 8,013 younger). The same inclusion and exclusion criteria were used for both older and younger participants. Some (66 older and 213 younger) were removed from the analysis because the computer system failed to randomize them within 24 h, allowing them to continue with the Lumosity program in the free user state. Following randomization (which was independent of age), the cognitive training group consisted of 5,051 participants (1,087 older and 3,964 younger) and the crosswords control group consisted of 4,868 participants (1,032 older and 3,836 younger). The post-test battery was completed by 53% of the participants (70 older and 48% younger) in the cognitive training group and 49% of participants (61 older and 46% younger) in the crosswords group. Additional participants (61 older and 269 younger) were removed from the crossword group because they were able to navigate around computer controls and complete some amount of cognitive training with Lumosity during the study period.

#### Demographics

The final fully evaluable sample in Hardy et al. (1) were divided into four groups comprising each combination of age cohort (younger vs. older) and training group (CCT vs. control). Table 1 shows the number of participants in each group, along with information about their age, gender, and level of education. While the quasi-experimental design resulted in more young than old participants, age cohort and training group were nearly orthogonal with respect to number of participants. That is, the proportions of old to young participants in the Lumosity (758:1909 = 1:2.52) and crosswords (570:1478 = 1:2.59) groups are nearly the same. Moreover, the two training groups within each age cohort were well-matched with respect to age mean and SD. Gender and educational level were also balanced across training groups within each age cohort, but not across the two cohorts. Younger participants were more likely to be male and have completed more years of education than older participants. How these differences between the two age cohorts influenced the relative size of their respective CCT effects is examined in the Results section.

**Table 1:** Number, age, gender, and educational level of participants in each age cohort and training group.

	**Younger Participants**			**Older Participants**			**All Participants**	
	**CCT**	**Control**	***p***	**CCT**	**Control**	***p***	**p Age Group**	**p Treatment**
N Participants	1909	1478		758	570			
Age (years)			0.88			0.13	<2.2e−16	0.67
Mean	30.83	30.77		59.39	58.66			
SD	8.64	8.58		6.60	6.66			
Gender (%)			0.46			0.84	<2.2e−16	0.55
Female	49.76	48.85		65.96	66.32			
Male	47.72	49.46		31.53	30.70			
Unreported	2.51	1.69		2.51	2.98			
Education (%)			0.44			0.73	0.00075	0.40
HS grad or less	10.16	10.76		14.25	12.81			
Some college	22.79	24.70		24.41	27.02			
Bachelor's degree	30.59	31.87		29.29	30.18			
^*^Advanced degree	30.38	28.48		25.33	25.26			
Unreported	6.08	4.19		6.73	4.74			

P values are based on Kolmogorov-Smirnov tests for age, chi-square for gender and education. Statistics for gender and education are based on participants who reported this information.

* Masters, Ph.D., or professional degree.

#### Compliance

The measure of compliance examined here was the primary one employed by Hardy et al. (1). As participants were instructed and reminded via email to complete daily sessions, they chose to use the number of active days of training. The younger participants trained on an average of 42.69 and 45.13 days, respectively, in the control and CCT groups. The older participants trained on an average of 52.81 and 50.34 days, respectively, in the control and CCT groups. When averaged across age cohort, there was little difference in number of active days between the control and CCT groups [0.01 days, *F*_(1,4711)_ = 0.0007, *p* = 0.9792]. In contrast, when averaged across training group, older participants engaged in more training than younger ones [7.67 days, *F*_(1,4711)_ = 192.5462, *p* < 0.001]. There was also a significant crossover interaction between age and training groups [*F*_(1,4711)_ = 19.6634, *p* < 0.001]. While younger participants spent more days training in the CCT than control group (2.44 days), older participants engaged in more training in the control group (2.47 days). How this pattern of compliance influenced the pattern of CCT effects is examined in the Results section.

### CCT and Control Activities

Participants in the Hardy et al. (1) study were instructed to log into the Lumosity website and perform one session per day of their activity (Lumosity for the CCT group or crossword puzzles for the control group), 5 days a week for 10 weeks. Daily email reminders were sent to all participants during the training period.

#### Cognitive Training

Lumosity games are organized into five cognitive domains according to their primary cognitive demand: Memory, Attention, Flexibility, Problem Solving, or Speed. Although each game is given a single category label, the majority of games make multiple cognitive demands (e.g., processing speed and flexibility, or attention and memory). Daily training sessions included five games. On any given day, the five games for that particular session were chosen by an algorithm that attempted to optimize a balance of training activities, such that games were presented in clusters across days without repetition on the same day. A single five-game session typically took about 15 min to complete. Outside of the sessions, participants could opt to do additional training with any of the 49 available games in an a la carte fashion. A description of each game is provided in the S1 Appendix accompanying Hardy et al. (1).

#### Crossword Puzzles Control

The puzzles were produced by professional crossword puzzle constructors and presented in a website frame designed to replicate the look and feel of that for the CCT. Constructors were asked to create puzzles of medium difficulty, approximately equivalent to those presented in the New York Times on Thursdays (NYT crossword puzzles increase in difficulty throughout the week, with the most difficult on Saturdays). Participants typed their answers in the appropriate boxes and received feedback immediately following submission of a completed puzzle. In each daily session, participants were instructed to complete as many puzzles as possible within 15 min. If they completed a puzzle within the allotted time, the crossword application would provide a new one. After 15 min, participants were able to continue to work on the current puzzle for as long as they chose but were not given additional ones that day.

### Outcome Measures

#### NeuroCognitive Performance Test

The NCPT (32) is a brief, repeatable, web-based cognitive assessment platform. The specific battery used in the Hardy et al. (1) study took between 20 and 30 min to complete and included the seven subtests described below (Further details can be found in the S2 Appendix of Hardy et al.):

1) Forward Visual Memory Span and 2) Reverse Visual Memory Span: Based on the Corsi Blocks tasks (33) and designed to assess visual short-term and working memory, respectively. Participants were required to recall a sequence of randomized spatial locations in either forward or reverse order.
3) Grammatical Reasoning: Based on Baddeley's (34) Grammatical Reasoning Test and designed to assess cognitive flexibility and reasoning. This subtest required participants to rapidly and accurately evaluate potentially confusing grammatical statements.
4) Progressive Matrices: Based on established matrix reasoning assessments (35) and designed to assess problem solving and fluid reasoning.
5) Go/No-Go: Designed to assess response inhibition and processing speed. Participants were required to respond as quickly as possible to a target, but to avoid responding to distractors.
6) Arithmetic Reasoning: Designed to assess numerical problem solving ability (36). Participants were required to respond as quickly and accurately as possible to arithmetic problems written in words (e.g., “Four plus two =”).
7) Two-Target Search: Based on the Useful Field of View Test (37) and designed to measure divided visual attention. Participants were required to recall the locations of briefly presented target letters while ignoring distractors. This subtest was created for the purposes of the Hardy et al. study.

#### NCPT Scaling Procedure

As in the original Hardy et al. (1) study, performance on each subtest of the NCPT battery was scaled using a rank-based inverse normal transformation. Briefly, this involved comparing each participant's score on each subtest with a normative table for that subtest. Each normative table consisted of the rank-ordered pre-test scores on a single subtest of all participants who took both the pre- and post-tests. Based on the percentile of the equivalent score in the relevant normative table, each participant's score on a subtest was converted to a value in a normal distribution with a mean of 100 and SD of 15. Note that, unlike Hardy et al., separate tables for a subtest were not constructed for different age bins (as this would have removed performance differences between age cohorts). The sum of the scaled subtest scores for each participant was then used to generate their overall scaled score (the Grand Index) on the pre-test and on the post-test using a similar inverse normal transformation. See Morrison et al. (32) for a more detailed description of this procedure.

#### Self-Reported Outcomes

Immediately after finishing the NCPT (pre- and post-training), participants completed a survey (see Supplementary Datasheet 1) including nine questions related to specific cognitive failures (38) and successes as well as emotional status. Four questions concerned cognitive performance over the past month. For these, participants were asked to rate how often they: 1) lost track of details, 2) misplaced items, 3) lost concentration, and 4) remembered names. Response options were on a Likert scale: “Never,” “1–2 times during the month,” “1–2 times per week,” “Several times per week,” “Almost every day,” or “N/A.” The remaining five questions were about cognitive performance and emotional status over the past week. For these, participants were asked to rate their level of agreement with statements about whether they: 1) felt creative, 2) had good concentration, 3) felt anxious, 4) were in a bad mood, and 5) felt sad. Response options were on a Likert scale: “Strongly disagree,” “Disagree,” “Neither agree nor disagree,” “Agree,” “Strongly agree,” or “N/A.” Responses to all nine questions (except N/A) were recoded as 1–5, with 1 always signifying the most negative option. To calculate an overall survey score (Aggregate Survey Rating), analogous to the NCPT Grand Index, each participant's numeric responses to the nine questions were then averaged.

### Statistical Analyses

All the analyses reported in this study were performed using version 4.0.0 of the R statistical program (39). The dataset on which these analyses were performed is provided in Supplementary Datasheet 4. Descriptions of the variables and information about which were involved in each analysis are provided in Supplementary Datasheet 5. The information in these two sections should be sufficient to replicate the reported analyses (as well as perform additional ones).

#### Primary Outcomes and Analyses

Our primary goal was to compare the two age cohorts with respect to the overall effects of CCT. These overall effects were measured by the NCPT Grand Index and Aggregate Survey Rating. A two-way ANOVA was performed on each. The dependent variable was the change in either the NCPT Grand Index or Aggregate Survey Rating following training (assessment post-training minus assessment pre-training). The independent variables were Age (older vs. younger cohort) and Treatment (Lumosity training vs. crosswords training). The main effect of Treatment tests for the presence of a CCT effect averaged across the two age cohorts. The main effect of Age tests for a difference in change on the dependent measure (averaged across the Lumosity treatment and crosswords control groups). A comparison of the CCT effect between age cohorts is provided by the interaction between Treatment and Age.

##### Differences in Baseline

Directly comparing the size of CCT effects for older and younger participants presents a number of challenges. Included are baseline differences between the two age cohorts that might influence these comparisons or their interpretation. For example, the degree to which underlying cognitive functions can be improved might depend on their initial level, the outcome scores might butt up against boundaries of their limited ranges (ceiling and floor effects), or the relation between underlying functions and outcome measures might not be linear. Besides the possible presence of systematic differences in baseline between age cohorts, random variations within individual participants could also influence their baselines. By contributing to regression to the mean of outcome measures following training, this type of baseline variability could decrease sensitivity to the effects of CCT in general. To control for the effects of baseline differences between the age cohorts, as well as regression to the mean, we performed ANCOVAs in which baseline scores served as the covariate.

#### Secondary Outcomes and Analyses

A secondary goal was to compare the two age cohorts with respect to the effects of CCT on the individual NCPT subtests and survey items. This was accomplished by performing three-way mixed model ANOVAs on change scores for the subtests and change scores for the survey items. Age and Treatment served as between-subject factors, while Subtest (one level for each of the seven) or Item (one level for each of the nine) served as a within-subject factor. Of particular interest was the three-way interaction in each ANOVA, which evaluated whether differences between age cohorts in the effects of CCT (two-way Treatment × Age interactions) depended on the particular subtest or survey item. This interaction can be expressed also as a comparison between age cohorts on their profiles of CCT effects across subtests or items. By comparing their respective profiles, we sought to determine whether CCT produced qualitatively similar effects on the older and younger participants.

#### Controling for the Different Number of Old and Young Participants

Age was not a consideration in the original Hardy et al. (1) study. As a result, the 50 YO dividing line between the two age cohorts in our reanalysis of that study yielded a complete analysis of 1,328 older and 3,387 younger participants. Imbalances like this can sometimes cause factors to be non-orthogonal, which can bias results of ANOVAs or ANCOVAs employing Type I sums of squares (SS). In the present case, however, all factors are close to orthogonal because the proportions of old to young participants in the Lumosity (1:2.52) and crosswords (1:2.59) groups are nearly the same. Nonetheless, we adopted a common remedy for non-orthogonal factors, which was to employ Type III SS. To the extent that the factors are orthogonal, both SS types should produce equivalent results. Moreover, the size and significance of the highest interaction in an ANOVA or ANCOVA is unaffected by the type of SS, and this interaction was the focus of our primary (Age × Treatment) and secondary (Age × Treatment × Subtest or Item) analyses.

## Results

### Primary Outcomes and Analyses

#### Comparison of CCT Effects Between Age Cohorts

**Figure 1:**
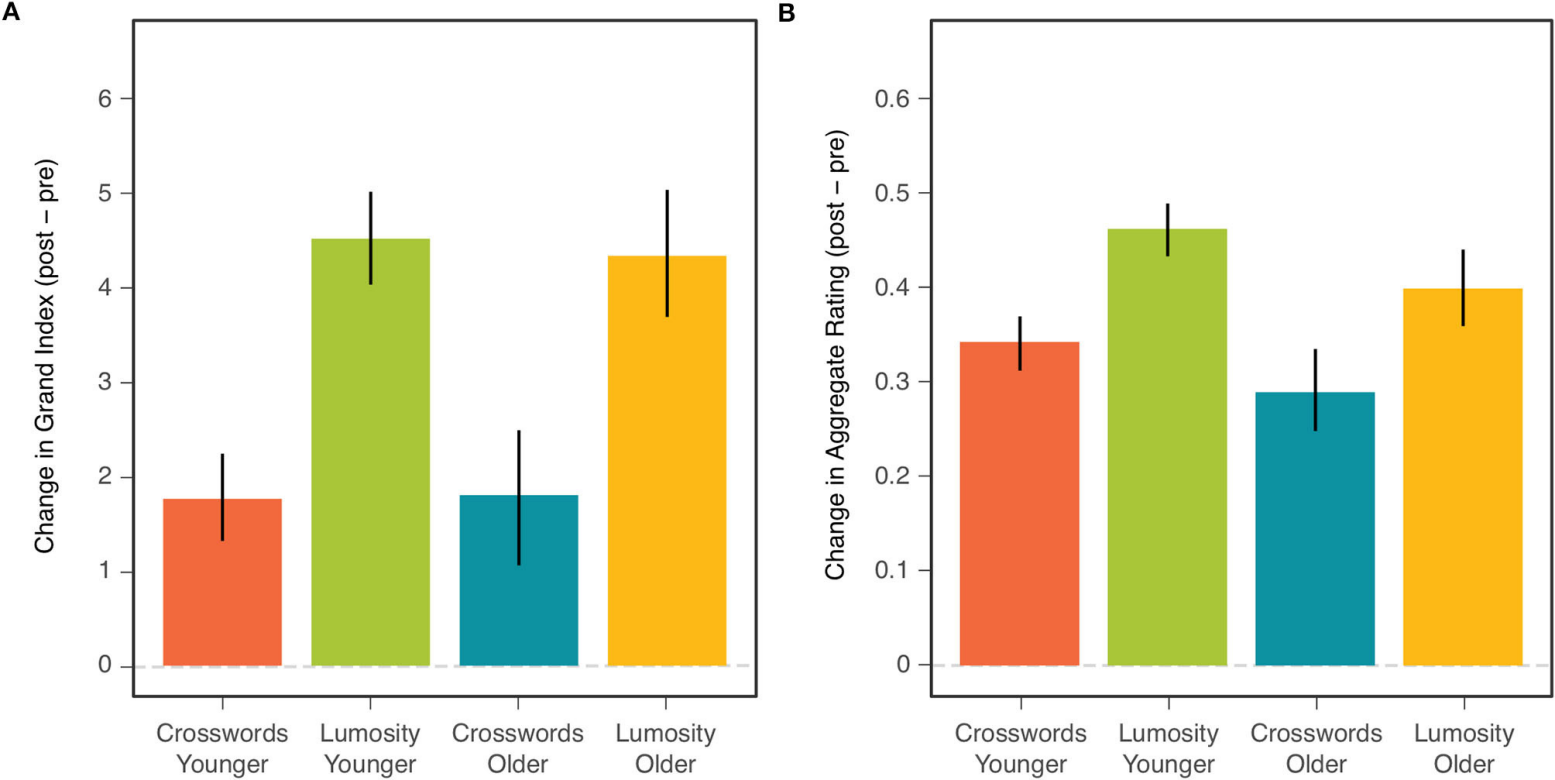
Mean change (post-pre) in NCPT Grand Index **(A)** and Aggregate Survey Rating **(B)** for each combination of age cohort and training group. Bars indicate 95% confidence intervals of the means.

Our primary goal was to compare the two age cohorts with respect to the overall effects of CCT. Such a comparison is displayed in Figure 1 for both the NCPT Grand Index (left) and Aggregate Rating on the survey (right). The effect of CCT corresponds to the difference in change (post- minus pre-training) score between the treatment (Lumosity) and control (crosswords) groups. The size of this effect on both measures was similar for younger (two leftmost bars in each panel) and older (two rightmost bars) adults.

Comparisons of the CCT effect between age groups correspond to the Age × Treatment effects shown in the ANOVA tables for the NCPT Grand Index (Table 2A) and Aggregate Survey Rating (Table 2B). This effect is far from significant for either measure (both F's <1). Also shown in each ANOVA table are the main effects of CCT (Treatment) and Age Cohort. Consistent with the analyses of Hardy et al. (1) on the combined age cohorts, the main effect of CCT on the change scores was highly significant for both measures. The main effect of age (on the combined change scores for both training groups) was significant for the Aggregate Survey Rating (young > old), but not for the NCPT Grand Index (F < 1).

**Table 2:** ANOVA results showing the effects of Age Cohort and Treatment on change (post-pre) in the NCPT Grand Index **(A)** and Aggregate Survey Rating **(B)**.

**Source**	**Type III Sum of Squares**	**df**	**Mean Square**	**F**	***p***
**(A) Change in NCPT Grand Index**					
Intercept	36097	1	36097	375.4500	<2e−16^***^
Age Cohort	5	1	5	0.0501	0.8229
Treatment	6529	1	6529	67.9087	<2e−16^***^
Age × Treatment	11	1	11	0.1181	0.7311
Error	452933	4711	96.1437		
**(B) Change in Aggregate Survey Ratings**					
Intercept	518.53	1	518.53	1497.6667	<2e−16^***^
Age Cohort	2.97	1	2.97	8.5785	0.0034^**^
Treatment	12.02	1	12.02	34.7096	4.095e-09^***^
Age × Treatment	0.03	1	0.03	0.0727	0.7875
Error	1623.11	4688	0.35		

*** p <0.001,

** p <0.01.

##### Descriptive Statistics

Table 3 presents descriptive statistics for both the NCPT Grand Index and Aggregate Ratings. Included for each combination of age cohort and training group are 1) baseline measures, 2) change scores, and 3) effect sizes (Cohen's d) for the change scores. Shown also for each age cohort is the difference in each measure between the two training groups (Lumosity - crosswords). As might be expected, baseline performance on the NCPT was lower for the older than younger participants. Despite this, the change scores and effect sizes for both age cohorts were similar in each training group, consistent with the lack of significant Age and Age × Treatment effects. Surprisingly, the baseline Aggregate Ratings on the survey were higher for older than younger participants. Consistent with the main effect of Age on this measure, the change scores and effect sizes in both training groups were larger for the younger than older participants. Yet, consistent with the lack of a significant Age × Treatment effect, the training group difference in change score and effect size was similar for both age cohorts.

**Table 3:** Descriptive statistics on baseline and change (post–pre) on the NCPT Grand Index and Aggregate Survey Rating for each age cohort and training group.

	**Crosswords (C)**		**Lumosity (L)**		**Training Group Difference** **(L – C)**	
	**Young**	**Old**	**Young**	**Old**	**Young**	**Old**
**NCPT**						
Baseline (points) Mean (SE)	104.54 (0.36)	90.22 (0.51)	103.44 (0.31)	88.04 (0.48)	−1.10 (0.473)	−2.18 (0.703)
Change (points) Mean (SE)	1.77 (0.24)	1.80 (0.35)	4.52 (0.24)	4.33 (0.35)	2.75 (0.342)	2.53 (0.500)
Effect Size (change) Cohen's d (95% CI)	0.189 (0.138 0.241)	0.213 (0.130 0.296)	0.428 (0.381 0.475)	0.447 (0.372 0.522)	0.274 (0.206 0.342)	0.275 (0.166 0.385)
**Survey**						
Baseline (rating) Mean (SE)	3.05 (0.015)	3.21 (0.024)	3.09 (0.013)	3.21 (0.023)	0.04 (0.020)	−0.01 (0.033)
Change (rating) Mean (SE)	0.34 (0.015)	0.29 (0.022)	0.46 (0.014)	0.40 (0.020)	0.12 (0.021)	0.11 (0.03)
Effect Size (change) Cohen's d (95% CI)	0.583 (0.528 0.639)	0.559 (0.470 0.647)	0.745 (0.694 0.796)	0.711 (0.632 0.792)	0.196 (0.128 0.265)	0.199 (0.090 0.308)

##### Confidence Intervals

Given the similar sizes of CCT effects observed in the two age cohorts (Table 3), the null hypothesis of no difference cannot be rejected for either the Grand Index or Aggregate Rating (Table 2). But what about alternative hypotheses involving non-zero differences? To evaluate a range of hypotheses, we calculated 90% confidence intervals for four statistics, each involving a different measure of CCT. The choice of 90% results in an alpha of *p* < 0.05 for each of two one-sided tests: one for values greater than the upper bound and the other for values less than the lower bound (40). Each statistic was the percent difference in CCT between the old and young relative to the young [((CCT(Old) – CCT(Young))/CCT(Young)) × 100], where CCT could be the training group difference in 1) mean change score or 2) Cohen's d on the 1) Grand Index or 2) Aggregate Rating. Note that positive values indicate larger CCT effects for the older participants.

The confidence intervals were generated using a bootstrap procedure (boot and boot.ci functions in the boot package for the R statistical program). The statistic involving change score on the Grand Index had a mean of −8.01%, lower bound of −40.86%, and upper bound of 32.41%. That involving Cohen's d for the Grand Index had a mean of 0.53%, lower bound of −34.41%, and an upper bound of 45.47%. The statistic involving change on the Aggregate Rating had a mean of −8.75% and ranged from −52.41 to 52.58%. That for Cohen's d had a mean of 1.48% and ranged from −46.73 to 68.21%. These confidence intervals allow us to reject extreme differences between the two age cohorts in the size of their respective CCT effects. But they do include differences of a meaningful size, albeit involving both lesser and greater benefits for the old relative to the young.

#### Controling for Differences in Demography and Compliance

##### Demographics

To what extent might the general pattern of effects observed in the above ANOVAs (Table 2) be due to demographic differences between older and younger participants in gender and educational level (Table 1)? Specifically, could these demographic differences, via their own effects on the efficacy of CCT, have obscured differences between the two age cohorts in the magnitude of CCT effects? To control for this possibility, four-way ANOVAs involving Age Cohort, Treatment, Gender, Educational Level, and all interactions were performed on both the NCPT Grand Index and Aggregate Survey Ratings.

Because these ANOVAs involved Type III sums of squares, each main effect and interaction was evaluated after the variance accounted for by all other main effects and interactions was removed from the model. Nonetheless, the pattern of age and treatment effects remained the same: The main effect of Treatment was still significant for both the Grand Index [*F*_(1,4381)_ = 60.7772, *p* < 0.001] and the Aggregate Ratings [*F*_(1,4363)_ = 27.8919, *p* < 0.001]; The main effect of Age Cohort remained non-significant for the Grand Index [*F*_(1,4381)_ = 0.1246, *p* = 0.7241] and significant for the Aggregate Ratings [*F*_(1,4363)_ = 9.8132, *p* = 0.0017]; The interaction between Treatment and Age Cohort remained non-significant for both the Grand Index [*F*_(1,4381)_ = 0.5521, *p* = 0.4575] and Aggregate Ratings [*F*_(1,4363)_ = 0.3508, *p* = 0.5537]. Full details about these ANOVAs and all their constituent effects are provided in Supplementary Datasheet 2.

##### Compliance

As reported in the section on compliance (Methods), 1) older participants trained on more days than younger ones and 2) while younger participants spent more days training on Lumosity than crosswords, the reverse was found for older participants. Was there an effect of number of training days on change in the NCPT Grand Index or Aggregate Survey Rating? If so, how did the pattern of compliance across training groups and age cohorts influence the pattern of CCT effects? To examine and control for the influence of compliance, ANCOVAs were performed on the NCPT and survey change scores. Treatment and Age served again as factors, and each participant's number of training days was included as a covariate.

When added as a covariate in ANCOVAs, number of active days did influence change scores on the NCPT Grand Index [*F*_(1,4710)_ = 20.9071, *p* < 0.001] and Aggregate Survey Rating [*F*_(1,4687)_ = 82.2033, *p* < 0.001]. However, the patterns of significance found for Treatment, Age Cohort, and their interaction remained the same as in the ANOVAs (Table 2). The main effect of Treatment remained significant for both the NCPT Grand Index [*F*_(1,4710)_ = 68.2244, *p* < 0.001] and Aggregate Survey Rating [*F*_(1,4687)_ = 35.6432, *p* < 0.001]. The main effect of Age Cohort was still significant for the survey [*F*_(1,4687)_ = 22.0734, *p* < 0.001] and still non-significant for the NCPT [*F*_(1,4710)_ = 1.2677, *p* = 0.2603]. Most importantly, the interaction between Age Cohort and Treatment remained non-significant for both the NCPT [*F*_(1,4710)_ = 0.0024, *p* = 0.9610] and survey [*F*_(1,4687)_ = 0.1017, *p* = 0.7498]. Thus, after controling for differences between age cohorts in compliance, the difference in size between their respective CCT effects remained non-significant.

#### Influence of Baseline on CCT Effects

**Figure 2:**
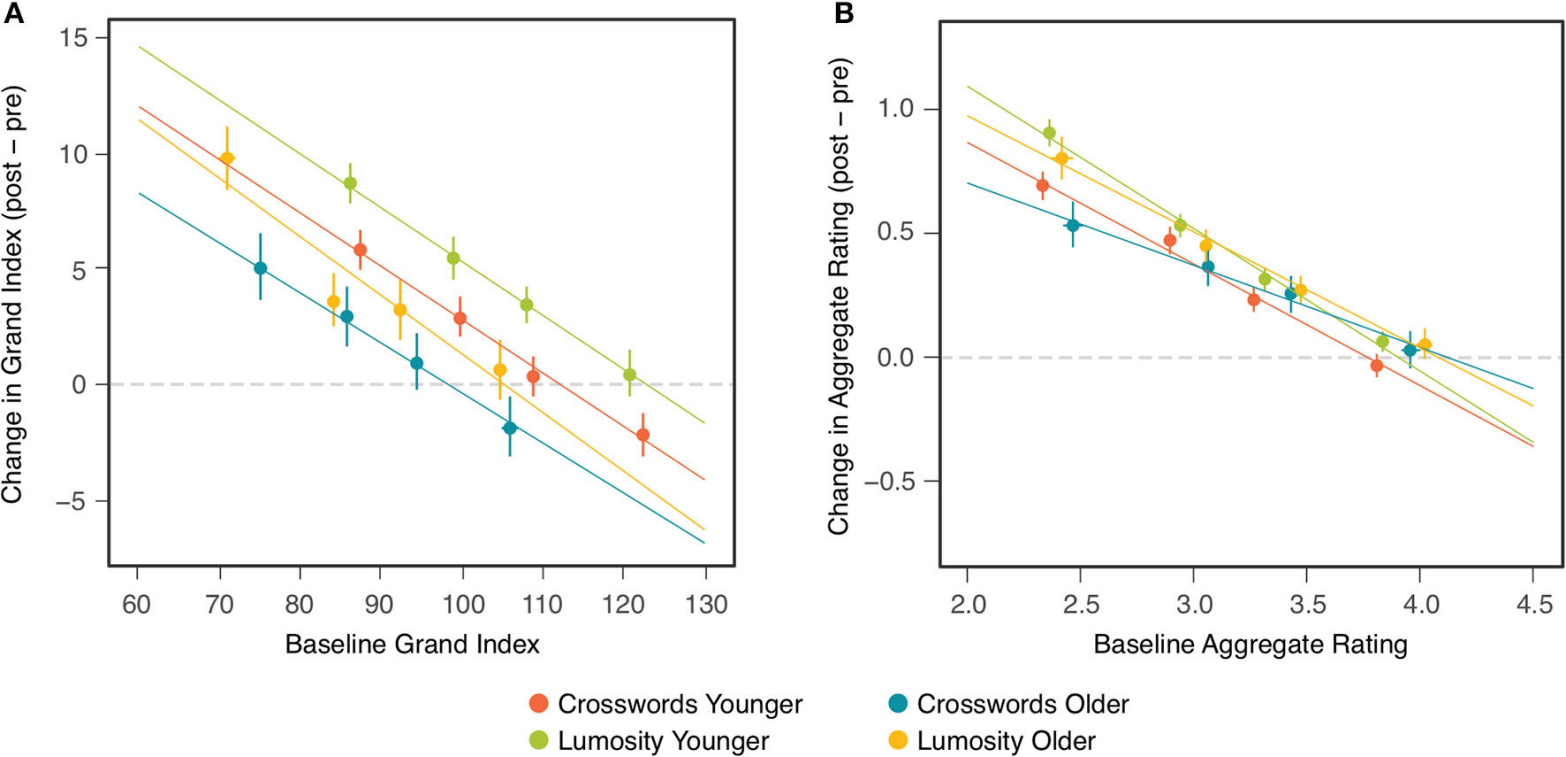
Change (post-pre) in NCPT Grand Index **(A)** and Aggregate Survey Rating **(B)** as a function of baseline assessment. Each panel includes four colors corresponding, respectively, to each combination of age cohort and training group. Within each combination, markers indicate the means of participants in quartile bins. Bars indicate 95% confidence intervals of the means. Lines show the fits of linear regression to the points for individual participants in each of the four combinations.

Comparisons of the CCT effects between the two age cohorts found no significant differences on either outcome measure. The results of these comparisons could have been influenced, however, by pre-existing differences between the two cohorts that affect change scores in ways unrelated to any cognitive benefits of CCT. One such factor is the baseline differences observed between older and younger participants [Table 3; see also (32)]. Figure 2 shows that change scores for the individual participants were inversely related to their baseline scores in both age cohorts, in both training groups, and on both outcome measures. This relation could have been due, at least in part, to ceiling and/or floor effects, which, given their different average baselines, might bias change-score measurement for the two cohorts in opposite directions. It can be seen also in Figure 2 that some of the highest baseline scores are associated with negative change scores, which suggests the presence of regression to the mean. To control for the effects of baseline differences between the age cohorts, as well as regression to the mean, the ANOVAs examining age and treatment group (Table 2) were repeated as ANCOVAs in which each participant's baseline measure was included as a covariate.

**Figure 3:**
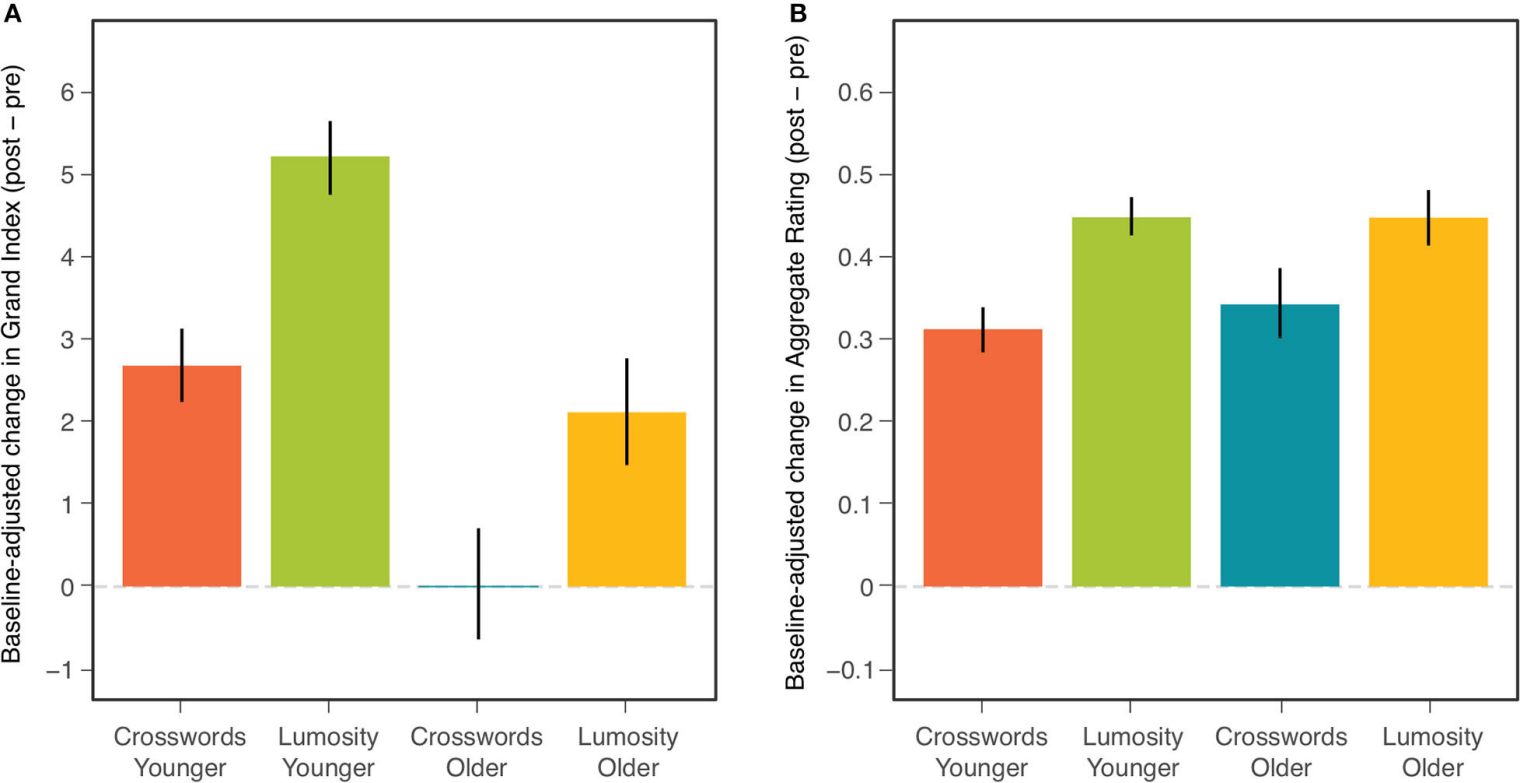
Mean baseline-adjusted change (post-pre) in NCPT Grand Index **(A)** and Aggregate Survey Rating **(B)** for each combination of age cohort and training group. Bars indicate 95% confidence intervals of the means.

Table 4 presents results of these ANCOVAs for both the NCPT Grand Index (Panel A) and Aggregate Survey Rating (Panel B). The effects of baseline on change score can be seen here to be highly significant for both measures. Nonetheless, after controling for these baseline effects, 1) the overall effect of CCT (Treatment) remained highly significant for both measures and 2) the difference in the CCT effect between age cohorts (Age × Treatment) remained non-significant for both measures. Controling for individual differences in baseline did, however, produce differences in the main effect of Age Cohort on the change scores. This effect changed from non-significant to significant for the NCPT Grand Index and from significant to non-significant for the Aggregate Rating (compare with Table 2). These effects of Age are considered further in the Discussion. The Age, Treatment, and Age × Treatment effects are displayed graphically in Figure 3 (compare with Figure 1).

**Table 4:** ANCOVA results showing the effects of Age Cohort and Treatment on baseline-adjusted change (post-pre) in the NCPT Grand Index **(A)** and Aggregate Survey Rating **(B)**.

**Source**	**Type III Sum of Squares**	**df**	**Mean Square**	**F**	***p***
**(A) Change in NCPT Grand Index**					
Intercept	58851	1	58851	682.1857	<2.2e−16^***^
Baseline (covariate)	46610	1	46610	540.2955	<2.2e−16^***^
Age Cohort	9507	1	9507	110.1996	<2.2e−16^***^
Treatment	4752	1	4752	55.0850	1.362e−13^***^
Age × Treatment	52	1	52	0.6081	0.4355
Error	406323	4710	86.27		
**(B) Change in Aggregate Survey Ratings**					
Intercept	598.89	1	598.89	2310.7787	<2.2e−16^***^
Baseline (covariate)	408.37	1	408.37	1575.6572	<2.2e−16^***^
Age Cohort	0.22	1	0.22	0.8301	0.3623
Treatment	13.55	1	13.55	52.2863	5.575e−13^***^
Age × Treatment	0.22	1	0.22	0.8527	0.3558
Error	1214.74	4687	0.26		

*** p <0.001.

#### Analyses Involving Three Age Cohorts

Finally, we sought to determine whether the results of the primary analyses, in particular the lack of a significant interaction between Age and Treatment, could have been due to the number or definition of age cohorts. Perhaps age differences in the effects of CCT were obscured by our having employed only a younger and older cohort divided at 50. To examine this possibility, we performed additional analyses employing an alternative age factor comprised of three cohorts: young (18–39), middle aged (40–64), and old (65–80). The three-cohort Age and Treatment factors were included in ANOVAs on the Grand Index and Aggregate Survey Rating, as well as in the corresponding ANCOVAs that controlled for baseline differences between participants.

The same patterns of effects were found for the three-cohort Age factor as in the analyses involving the two-cohort Age factor. Treatment was significant in the ANOVAs and ANCOVAs for both dependent measures. The effect of Age on change score was non-significant in the ANOVA and significant in the ANCOVA for the Grand Index; the reverse was found for the Aggregate Survey Rating. Importantly, the Age × Treatment interaction was non-significant in all four analyses. ANOVA and ANCOVA tables reporting these statistics, along with further information on the three age cohorts, are provided in Supplementary Datasheet 3.

### Secondary Outcomes and Analyses

Our secondary goal was to compare the two age cohorts with respect to the effects of CCT on the individual NCPT subtests and survey items. For purposes of description, we calculated confidence intervals for the effects of Treatment, Age Cohort, and their interaction on the change scores for each separate subtest and item. To evaluate these effects statistically, three-way mixed model ANOVAs were performed, respectively, on change scores for the individual subtests and survey items. Age Cohort and Treatment served as between-subject factors, while Subtest (one level for each of the seven) or Item (one level for each of the nine) served as a within-subject factor. Of especial interest was the three-way interaction (Treatment × Age × Subtest/Item) in each ANOVA, which evaluated whether differences between age cohorts in the effects of CCT (Treatment × Age interaction) depend on the particular subtest or survey item.

**Figure 4:**
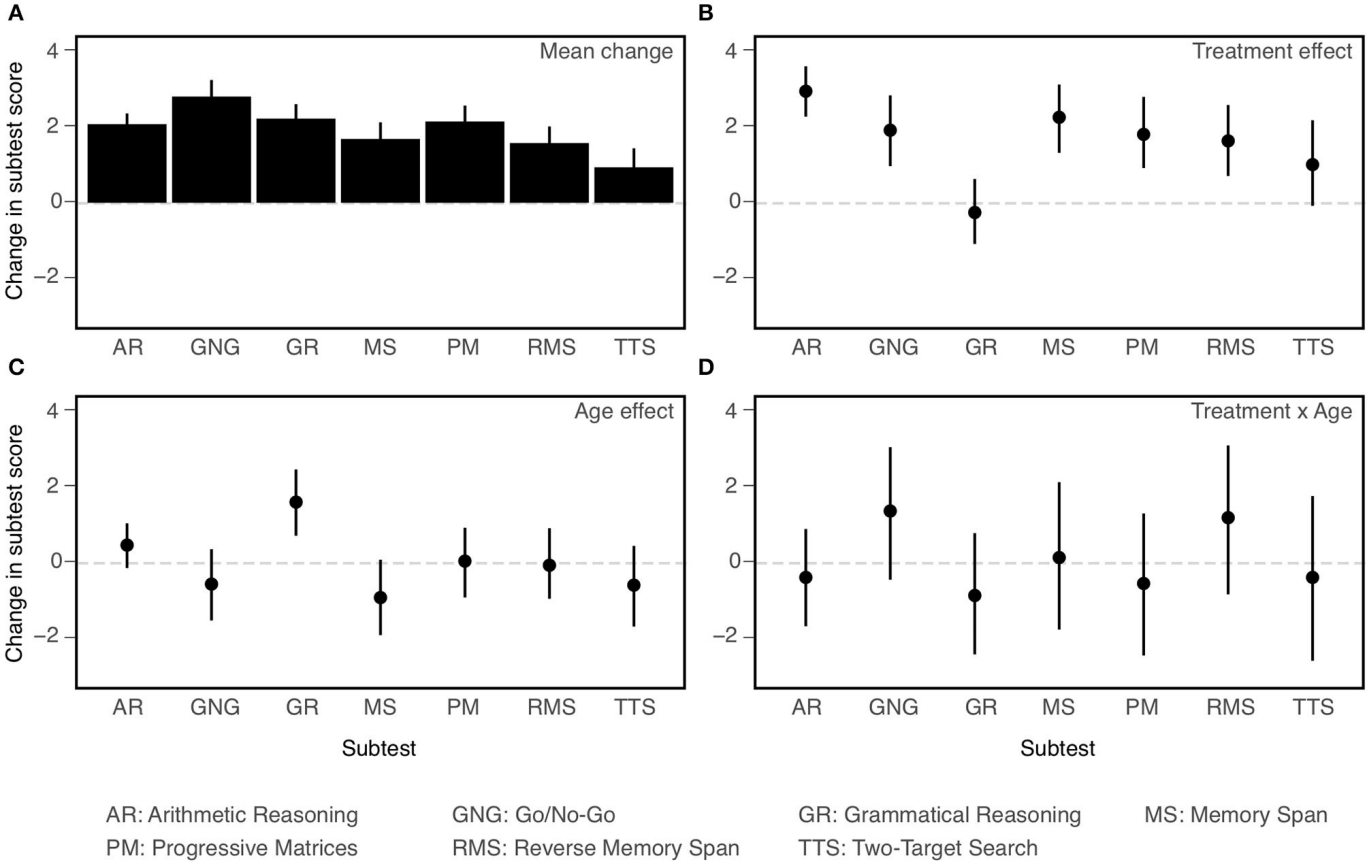
Effects on change (post-pre) in performance on individual NCPT subtests (all coded so that larger values indicate better performance). Mean change averaged across age cohorts and training groups **(A)**; Main effect of Treatment **(B)**; Main effect of Age **(C)**; Treatment × Age interaction **(D)**. Bars indicate 95% confidence intervals of the mean.

Let us consider first the individual NCPT subtests. Confidence intervals on effect sizes for each are shown in Figure 4 and ANOVA results are presented in Table 5. A number of conclusions suggested visually in the figure are supported by the ANOVA. Panel A of the figure displays the overall change between the first and second assessments averaged across age cohorts and training groups. The main effect of Subtest in the ANOVA shows that the size of the change differed significantly across subtests. Panel B displays the CCT effect averaged across the two age cohorts. The main effect of Treatment shows that the CCT effect was significant overall, and the Treatment × Subtest interaction shows that it varied significantly in size across subtests. Panel C displays the differences in change score between the two age cohorts averaged across training groups. The main effect of Age and Age × Subtest interaction show that, while there was no significant effect of age overall, its effect on change score did vary significantly across subtests. Finally, Panel D displays the differences in CCT effects between the two age cohorts. The non-significant Treatment × Age and Treatment × Age × Subtest interactions show that, not only did the two age cohorts not differ significantly in the size of their overall CCT effect, but also in the sizes of their CCT effects on all the individual subtests.

**Table 5:** Mixed-model ANOVA results showing the effects of Age Cohort, Treatment, and Subtest on change (post-pre) in the NCPT.

**Predictor**	***df_***Num***_***	***df_***Den***_***	***Epsilon***	***SS_***Num***_***	***SS_***Den***_***	***F***	***p***
(Intercept)	1.00	4711.00		89030.77	1105345.91	379.45	0.000
Treatment	1.00	4711.00		16290.85	1105345.91	69.43	0.000
Age Cohort	1.00	4711.00		12.92	1105345.91	0.06	0.815
Treatment × Age	1.00	4711.00		1.80	1105345.91	0.01	0.930
Subtest	5.65	26611.78	0.94	7806.98	5870189.08	6.27	0.000
Treatment × Subtest	5.65	26611.78	0.94	5769.86	5870189.08	4.63	0.000
Age × Subtest	5.65	26611.78	0.94	4025.16	5870189.08	3.23	0.004
Treatment × Age × Subtest	5.65	26611.78	0.94	1077.51	5870189.08	0.86	0.515

df_Num_ indicates degrees of freedom numerator. df_Den_ indicates degrees of freedom denominator. Epsilon indicates Greenhouse-Geisser multiplier for degrees of freedom, p-values and degrees of freedom in the table incorporate this correction. SS = Type III. SS_Num_ indicates sum of squares numerator. SS_Den_ indicates sum of squares denominator.

**Figure 5:**
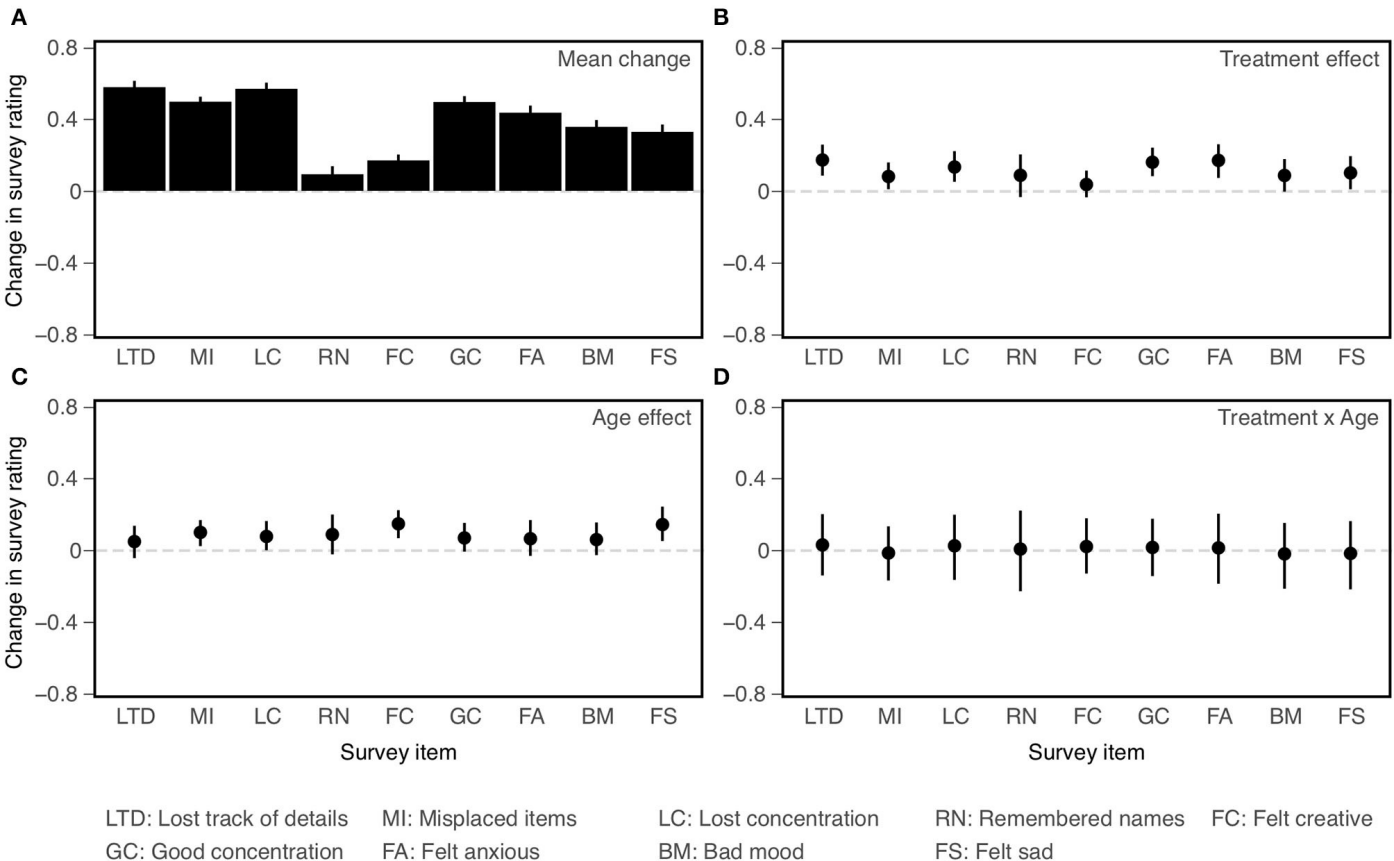
Effects on change (post-pre) in ratings on individual survey items (all coded so that larger values indicate more positive ratings). Mean change averaged across age cohorts and training groups **(A)**; Main effect of Treatment **(B)**; Main effect of Age **(C)**; Treatment × Age interaction **(D)**. Bars indicate 95% confidence intervals of the mean.

Confidence intervals on effect sizes for each survey item are shown in Figure 5 and ANOVA results are presented in Table 6. As with the NCPT subtests, overall change between the two assessments varied significantly across survey items (Panel A in figure, main effect of Item in table). The results concerning the effects of CCT (averaged across age cohorts) and age (averaged across training groups) differed somewhat from those found for the subtests. There was a significant effect of CCT on change scores (Lumosity > crosswords), but its size did not vary significantly across items (Panel B, main effect of Treatment and Treatment × Item interaction). Likewise, the effect of age was significant overall (young > old) but did not vary significantly across items (Panel C, main effect of Age and Age × Item interaction). As found for the subtests, the overall effects of CCT did not differ significantly between age cohorts, nor did the size of this difference vary significantly across items (Panel D, Treatment × Age and Treatment × Age × Item interactions).

**Table 6:** Mixed-model ANOVA results showing the effects of Age Cohort, Treatment, and Item on change (post-pre) in survey rating.

**Predictor**	***df_***Num***_***	***df_***Den***_***	***Epsilon***	***SS_***Num***_***	***SS_***Den***_***	***F***	***p***
(Intercept)	1.00	3389.00		3253.85	10375.41	1062.83	0.000
Treatment	1.00	3389.00		64.49	10375.41	21.07	0.000
Age Cohort	1.00	3389.00		36.41	10375.41	11.89	0.001
Treatment × Age	1.00	3389.00		0.01	10375.41	0.00	0.956
Item	6.91	23416.44	0.86	598.03	32099.39	63.14	0.000
Treatment × Item	6.91	23416.44	0.86	11.53	32099.39	1.22	0.289
Age × Item	6.91	23416.44	0.86	6.51	32099.39	0.69	0.681
Treatment × Age × Item	6.91	23416.44	0.86	0.48	32099.39	0.05	1.0

df_Num_ indicates degrees of freedom numerator. df_Den_ indicates degrees of freedom denominator. Epsilon indicates Greenhouse-Geisser multiplier for degrees of freedom, p-values and degrees of freedom in the table incorporate this correction. SS = Type III. SS_Num_ indicates sum of squares numerator. SS_Den_ indicates sum of squares denominator.

The main conclusions of the secondary analyses are as follows. First, overall changes in score or rating between the first and second assessments varied significantly across subtests and items. Second, results of the primary analyses were confirmed. Though based on somewhat different models, the ANOVAs employed in the primary and secondary analyses yielded the same pattern of main and interaction effects for Age and Treatment. Third, some of these effects vary across individual subtests or items, indicating a degree of selectivity. Both CCT (Treatment) and Age interacted with Subtest, but not with Item. Consistent with the findings of Hardy et al. (1), the effect of CCT was smaller for Grammatical Reasoning than the other subtests. Conversely, Grammatical Reasoning showed greater age effects (young > old) than did the other subtests. The final and most important conclusion is based on the lack of significant three-way interactions. Age differences in the CCT effect did not vary significantly across subtests or items. Equivalently, the two age cohorts did not differ significantly in their profiles of CCT effects across either subtests or items.

## Discussion

There has been considerable interest in using CCT to slow or remediate cognitive decline in older adults, both normal and pathological. Toward these ends, it would be useful to know how the effects of CCT on cognitive function vary over the course of normal cognitive aging. Are there changes in either the overall efficacy of CCT or in which cognitive faculties are affected? Here, we reanalyzed the results of a large online study by Hardy et al. (1) in order to compare the effects of CCT on younger vs. older adults. Our primary goal was to compare the size of the overall effects of CCT on each of two types of measure employed by Hardy et al.: performance on the neurocognitive test battery (NCPT) and self-reported ratings on a survey of cognition and affect in daily living. Our secondary goal was to compare the size of effects on the individual subtests of the battery and items on the survey, in order to examine whether CCT produced qualitatively similar effects on the two age cohorts.

### Comparison of CCT Effects on Older vs. Younger Participants

As in the Hardy et al. (1) study, effects of CCT were found on overall measures of the NCPT and survey. Change in the Grand Index and Aggregate Rating was significantly greater following training in the Lumosity treatment group than in the crosswords control group. But these effects did not differ significantly in size between older and younger participants. That is, no differences in overall size of CCT effects were found between the two age cohorts. This does not imply, however, that these CCT effects were statistically equivalent (40). Despite their minimal observed differences, the confidence intervals around these differences included ones sufficiently large to be considered meaningful.

To what extent were the results of comparisons between the two age cohorts influenced by differences in their respective assessment (pre-training) baselines? We observed for individual participants in both age cohorts and training groups that change scores on both outcome measures were inversely related to their baseline scores. While this relation may have resulted in part from regression to the mean, changes in cognitive function may also have depended on their initial level. Could there have been a similar relation between the mean baselines and CCT effects observed for each cohort? To control statistically for this possibility, the ANOVAs examining age and training group were repeated as ANCOVAs in which each participant's baseline measure was included as a covariate.

It should be acknowledged that there exists some controversy about using ANCOVA to control for pre-existing differences between study groups. For example, it has been argued that using ANCOVA to equate groups on such differences can inappropriately remove variance from the dependent variable (41). It can also be debated whether equating old and young participants on their assessment baselines makes them more or less cognitively commensurate [e.g., (42)]. But such concerns may be moot given that the same pattern, i.e., the presence of CCT effects and their lack of significant modulation by age, was found with and without controling for pre-existing differences in baseline between the two age cohorts.

Also relevant to comparisons between the age cohorts is how these cohorts were defined in our study. Perhaps age differences in the effects of CCT were obscured by our having employed only a younger and older cohort divided at 50. To examine this possibility, we performed additional analyses employing an alternative age factor comprised of three cohorts: young (18–39), middle aged (40–64), and old (65–80). Analyses were performed on both the Grand Index and Aggregate Rating, both with and without baseline correction. In each, the same pattern of effects was found for the three-cohort Age factor as in the corresponding analysis involving the two-cohort Age factor. From this we conclude that the absence of detectable age differences in overall CCT effects was not due to the number of cohorts or age cutoff between them.

Besides comparing their relative sizes, we sought to determine whether effects of CCT on the two age cohorts were mediated by identical mechanisms. Possible differences between the mechanisms might include the identities of the affected cognitive faculties or the pattern of relative influence across the same set of multiple faculties. But whatever they might be, qualitative differences in the effects of CCT on the two age cohorts might be expected to have resulted in different profiles of effect sizes across the individual NCPT subtests or survey items. Yet, no detectable differences were found between the effect profiles for old and young participants. That is, there was no significant three-way interaction between age cohort, treatment, and subtest or survey item. The absence of this three-way interaction can be viewed in two ways. One is that the effect size of CCT did not differ significantly between old and young participants for any of the individual NCPT subtests or survey items. The other view is in terms of the pattern of differences in CCT effect sizes across the set of subtests or items, which likewise did not differ significantly between the two age cohorts. These results support the conclusion that the old and young were influenced by CCT in the same way.

### CCT Effects on Older Adults

The absence of significant age differences in the effects of CCT may seem surprising given the neural and cognitive changes known to accompany normal aging (43). These include changes across much of the adult lifespan in the effects of practice on a wide variety of activities, including Lumosity games [e.g., (44)]. Indeed, a number of effects in the current study were also consistent with diminished learning ability in the older participants: A significant main effect of age was found on change in 1) NCPT Grand Index after baseline adjustment and 2) Aggregate Survey Rating prior to baseline adjustment. In each case, greater change in a positive direction was found for younger than older participants. These effects, however, involve change averaged across the treatment and control groups. Despite these overall age differences, age did not significantly influence the degree to which change in the Lumosity group exceeded that in the crosswords group.

Regardless of how they compare to younger adults, the amount by which older adults benefited from CCT is encouraging news for combatting the negative effects of cognitive aging. While in agreement that CCT may benefit older adults, some have expressed skepticism about the efficacy of commercial programs delivered over the internet (5). Our findings support a more optimistic view of remote training. As in the Corbett et al. (25) study, which likewise delivered CCT over the internet (some of which closely resembled commercial programs), we found positive effects for older adults on both neuropsychological tests and self-ratings of everyday cognition. The effect sizes in both studies on both types of measure were in the small to medium range. This is comparable to those found in large studies of CCT in the clinic.

This does not imply that CCT is as effective without any in-person social, instructional, or therapeutic interaction. Such interactions may be especially important in cognitive rehabilitation in order to provide intensive CT following acquired brain damage [e.g., (45)]. They are also key features of the CT protocol in many studies examining its impact on cognitive aging (e.g., ACTIVE and IHAM). However, delivery of CCT over the internet could potentially serve as a valuable addition. Besides improving access and reducing the cost of CT for older adults, it makes more practicable the practice of CT over an extended period. Such long-term practice could help maintain cognitive function and possibly yield larger effects or more distant transfer than the shorter interventions available at a clinic or lab. Future research should consider both efficacy and effectiveness in real-world conditions when exploring differences between at-home CCT and CT administered by a therapist.

### Nature of Training in the Current Study

The findings of this study suggest that the same cognitive faculties mediated CCT effects in the older participants as in the younger ones. But which faculties are these? While their specific identities are uncertain, there were clues about some of their general characteristics.

The effects of CCT on the NCPT and survey might be considered, respectively, as near and far transfer. Though the NCPT subtests are distinct from Lumosity games and vary in their degree of similarity to these games, their structure—i.e., brief, computerized cognitive tasks—resembles Lumosity games more than crossword puzzles. With the possible exception of grammatical reasoning, they are therefore more likely to assess those faculties engaged more by the former than the latter. And indeed greater effects were found for Lumosity than crosswords training on all subtests except grammatical reasoning (see Figure 4B). Because far transfer involves activities quite different from those trained, it is likely to be mediated by faculties that support a wide variety of activities. That some of the trained faculties contribute to everyday cognitive activities (and associated affective states) is suggested by the effects of CCT on the survey.

In general, any benefits that older individuals might receive from CT could involve either cognitive faculties that decline with age or non-declining faculties that can compensate for declining ones. The current findings suggest that benefits by the older participants on the NCPT were of the latter type. Specifically, while baseline performance on the NCPT declined with age [see also (32)], the effects of CCT on this measure were not significantly influenced by age. The decline in baseline with age indicates that at least some of the faculties that contribute to NCPT performance likewise decline with age. That the effects of CCT on the NCPT were independent of age, while not conclusive, suggests that they were not mediated by these particular age-sensitive faculties.

There are, however, cognitive faculties known to decline with age that were not assessed in the current study. Chief among these is long-term episodic memory, which is an important feature of ARCD, MCI, and dementia. Though we did find a positive effect of CCT in the survey on the self-reported incidence of forgetting names, none of the NCPT subtests directly assessed episodic memory. Other studies of cognitive aging (e.g., ACTIVE) have found beneficial effects of CCT on episodic memory. The effects of training with Lumosity in particular (vs. crossword puzzles) are currently under study in an RCT involving patients with MCI (29).

### Crossword Puzzle Control Group

How do the findings of this study and their interpretation depend on the choice of crossword puzzles as training for the control group? As is typical in RCTs with assessments before and after a treatment, the effects of CCT were estimated by subtracting the change scores of the control group from those of the treatment group. This subtraction was intended to remove the contribution to the change scores of what the two groups have in common, leaving the effect of their differences. Let us consider what the two groups had in common and how they differed.

Ideally, the treatment and control activities should be equated for any effects on the outcome measures observed in either group that were not due to CCT. One such effect in the present study could result from taking the NCPT or survey twice. A control group involving almost any activity (or none) would have been sufficient to remove this effect. More difficult to control in studies involving cognitive treatments are effects from expectations of improvement on the outcome measures, i.e., placebo effects, which have been found in some studies of CT (46, 47). Crosswords puzzles were chosen by Hardy et al. (1) for the active control group because they are popularly believed to be beneficial for cognition (48). Indeed, a recent study of public perceptions found similar levels of such expectations for crossword puzzles and brain training games (49).

There were quite a few differences between Lumosity and crosswords training. These included cross-training on diverse games vs. training on a single type of puzzle. The difficulty of many Lumosity games was also adjusted adaptively, while all crossword puzzles were of the same moderate difficulty. It cannot be determined which of these or other differences was responsible for the larger effect of Lumosity than crossword puzzle training on the NCPT and survey. While such information would help identify the “active ingredient(s),” it is not necessary for determining whether a valid effect of CCT was present. The situation here is analogous to that in the FINGER study (50) of older adults at risk for dementia, in which a single treatment group received an intervention combining CT, physical exercise, dietary guidance, and vascular monitoring. Greater benefit on a neuropsychological test battery was found in the treatment group than in a control group that received only general health advice. While the findings support the efficacy of a complex intervention, the relative contribution of its different components has yet to be determined.

Finally, it is preferable that a control activity not produce any bonafide CT effects of its own. There is, however, evidence that engaging in crossword puzzles can result in cognitive benefits beyond mere improvement on these puzzles. For example, Pillai et al. (51) examined memory in a group of older individuals who developed dementia during their participation in the longitudinal Bronx Aging Study. Greater self-reported engagement in crossword puzzles at study entry was associated with a delay in the precipitous decline of memory preceding dementia, though not in the onset of dementia itself. Similarly, examining older adults at entry into the PROTECT study of cognitive aging, Brooker et al. (52) found associations between self-reported engagement in word puzzles and performance on two test batteries assessing a wide range of cognitive functions. To the extent that there were genuine CCT effects from crosswords training in Hardy et al. (1), subtracting the change scores for the crosswords groups from those for the Lumosity groups would lead to an underestimate of CCT effects attributed to Lumosity training.

### Limitations

The limitations of the present study arise from two sources: the original study from which the data was obtained and our reanalysis of that data. Like most RCTs that examine effects of CCT, the Hardy et al. (1) study did not examine their long-term maintenance (5) or the possible placebo-like effects of expectations (46, 47). Also, as mentioned, training benefits in the crosswords control group may have biased (in a negative direction) the estimation of CCT effects.

Another possible source of bias was the high level of participant dropout (See Participants in Methods), which was greater for younger than older participants and for crossword puzzle than Lumosity training. Differences in dropout rate could have contributed to the differences in compliance found across the four age × training conditions. The pattern of compliance, however, was shown not to influence the pattern of CCT effects. But the possibility still remains that differences in ability between participants who stayed in or left the study could have biased estimation of CCT effects.

Among those limitations arising from our reanalysis of existing data are ones due to its quasi-experimental design. Sensitivity was diminished by the unequal number of participants in the two age cohorts. Fortunately, the proportion of old to young participants was nearly equal in the two training groups, enabling us to avoid complications that can result from an unbalanced design. Pre-existing differences between older and young participants, which led to differences in baseline measures and compliance, also proved to be challenging.

Other limitations concern assessment of the cognitive functions affected by ARCD. The NCPT subtests, all of which decline over the adult lifespan (32) assess multiple cognitive functions. But none directly assess an important constituent of cognitive aging: long-term episodic memory. The survey, while appropriate for the Hardy et al. (1) study, proved problematic for the examination of age differences. Though sensitive to the immediate effects of CCT, the difference in baseline found between the two age cohorts (old > young) is unlikely to have been an accurate reflection of their respective cognitive and affective states.

A final limitation is due to the age composition of the study sample. Though it did range from 18 to 80, the distribution is skewed toward the young. The size of the younger cohort is ~2.5 times that of the older cohort, and only about 6% of the participants (albeit a substantial 278) were 65 or older. A greater proportion of older participants would have facilitated analyses involving multiple older cohorts with narrower age ranges (see Supplementary Datasheet 3).

The above limitations suggest features that might be incorporated into future studies comparing the effects of CCT across different age cohorts. First, it would be desirable if the age factor could be included as part of the experimental design, thus allowing random selection of an equal number of participants in each age cohort. But even with such a design, it would probably be necessary still to grapple with pre-existing differences between the cohorts. More than two age cohorts, with some comprising only very old participants, would also be desirable. Additional features might include 1) an assessment of long-term episodic memory, 2) measures of expected improvement on the assessments administered before and after training, 3) a third set of assessments that followed training by as long a period as possible, 4) a control activity less likely than crosswords to produce cognitive benefits, 5) measures of cognition and/or affect in daily living known to be sensitive to ARCD, and/or 6) more effective strategies for the retention of participants.

## Conclusions

The findings of this study suggest that benefits from CCT can occur to a similar degree and in a similar way across an extended part of the adult lifespan. This conclusion is based on the lack of significant differences, either quantitative or qualitative, found between older and younger participants in the effects of CCT. These results extend both to performance on a neuropsychological test battery and ratings on a survey of cognition and affect in daily living. Moreover, though we cannot demonstrate their statistical equivalence (40), the similarity in CCT effects found for the two age cohorts is noteworthy.

Practically speaking, the amount by which older individuals benefit from CCT in and of itself may be more important than how they fare relative to younger individuals. The size of the CCT effects found here and in other large studies [e.g., ACTIVE, IHAMS, (25)] are small to medium. But even these are equivalent to several years of normal cognitive decline and can have important consequences for public health. That the effect sizes of CCT in our study and that of Corbett et al. are in the range found previously for older adults in studies that include in-person contact demonstrates that training remotely over the internet can, at the very least, serve as a useful addition. Moreover, this type of delivery makes long-term training much more practicable, which could potentially enhance benefits.

Our findings suggest that CCT influenced the same cognitive faculties for both older and younger participants. Are these faculties ones that decline with age or faculties that can compensate for declining ones? While far from definitive, our findings suggest the latter. The effects of age and CCT on both of our outcome measures appeared to be independent. That is, the effects of one factor did not depend on the level of the other. While this pattern of effects on the outcome measures does not necessarily imply that age and CCT affected completely different cognitive faculties, it would seem a more likely consequence than if the two factors affected any faculties in common. Perhaps, in contrast to the limited period of CCT examined here and in other RCTs, long-term practice by older adults would show clearer signs of strengthening declining faculties.

Though most directly applicable to normal cognitive aging, our findings pose an obvious question for studies examining CCT in groups with MCI or dementia. Both the size and qualitative profile of CCT effects may be relatively constant over a substantial portion of normal cognitive aging. If so, at what point does this change in the pathogenesis of dementias? Our findings highlight in particular the need to consider, not just the overall size, but also the qualitative pattern of CCT effects across outcome measures and their components. Qualitative differences from the normal pattern in patient groups could provide clues about changes in the mix of preserved trainable faculties, as well as help in the design of CCT that makes best use of this mix.

## Data Availability Statement

The dataset and description of variables can be found in Datasheets 4 and 5 in the Supplementary Material.

## Ethics Statement

This study, which solely involved the analysis of pre-existing de-identified data from human participants, met the criteria for exempt research. Ethical review and approval was not required on human participants in accordance with local legislation and institutional requirements. Written informed consent for participation was not required in accordance with national legislation and institutional requirements. The study in which the pre-existing data was obtained (1) was reviewed and approved by Ethical and Independent Review Services (IRB Protocol 13054-01). Written informed consent for all participants in this previously published study was obtained prior to their enrollment.

## Author Contributions

AO, KK, NN, and RS conceived the study and wrote the manuscript. AO, NN, and RS performed the data analyses. All authors participated in interpreting the de-identified data and editing the manuscript. All authors approved the submitted version.

## Conflict of Interest

The current study examined effects of cognitive training with Lumosity on the NeuroCognitive Performance Test, both of which are produced by Lumos Labs, Inc. AO, KK, NN, and RS are current employees and hold stock options in the company. PD is a former unpaid scientific advisor to Lumos and has received research access to Lumosity and the NeuroCognitive Performance Test for an ongoing NIH grant. PD has received grants and advisory or board fees from several other health and technology companies. PD owns stock in several companies whose products are not mentioned here and is a co-inventor on several patents that are not mentioned.
