# Supplementary material for: Computerized Cognitive Training by Healthy Older and Younger Adults: Age Comparisons of Overall Efficacy and Selective Effects on Cognition
Source: Front Neurol. 2021 Jan 8;11:564317. doi: 10.3389/fneur.2020.564317 (PMC7832391; doi:10.3389/fneur.2020.564317)
Supplement: Supplementary file 1 [file Data_Sheet_1.PDF]

Survey of Real-World Cognition and Affect

Immediately after finishing the NeuroCognitive Performance Test battery (both pre- and post-training), participants completed the survey shown below (Supplementary Figure 1). The first part of the survey (top panel) consisted of four questions concerning the frequency of cognitive successes or failures during the prior month. The second part (bottom panel) contained five questions about affective states related to cognition during the prior week. Responses to all nine questions involved selecting a point on a 5-point Likert scale (or "Not applicable").

1. During the PAST MONTH, how often have you...

	Never	1-2 times during the month	1-2 times per week	Several times per week	Almost every day	Not applicable
... lost track of details as you were reading and needed to go back and re-read sections?	○	○	○	○	○	○
... misplaced items (e.g., reading glasses, keys) around the house?	○	○	○	○	○	○
... found yourself losing concentration during a conversation?	○	○	○	○	○	○
... remembered someone's name who had just been introduced to you?	○	○	○	○	○	○

2. Over the LAST WEEK...

	Strongly disagree	Disagree	Neutral	Agree	Strongly agree	Not applicable
I felt creative.	○	○	○	○	○	○
My ability to concentrate was good.	○	○	○	○	○	○
I felt anxious.	○	○	○	○	○	○
I was in a bad mood.	○	○	○	○	○	○
I felt sad for no obvious reason.	○	○	○	○	○	○

Supplementary Figure 1. Rated items and response options on the survey of real-world cognition and affect.
