# Supplementary material for: Computerized Cognitive Training by Healthy Older and Younger Adults: Age Comparisons of Overall Efficacy and Selective Effects on Cognition
Source: Front Neurol. 2021 Jan 8;11:564317. doi: 10.3389/fneur.2020.564317 (PMC7832391; doi:10.3389/fneur.2020.564317)
Supplement: Supplementary file 2 [file Data_Sheet_2.pdf]

Controlling for Demographic Differences between Age Cohorts

Though gender and educational level were balanced across the treatment groups within each age cohort, they nonetheless differed across age cohort (Table 1 in article). Younger participants were more likely to be male and better educated than older participants. Could differences in gender and education, perhaps via their own influences on the efficacy of CCT, have obscured differences between the two age cohorts in the magnitude of CCT effects? To control for this possibility, four-way ANOVAs involving age cohort, treatment, gender, educational level, and all their interactions were performed on both the NCPT Grand Index (Supplementary Table 1) and Aggregate Survey Ratings (Supplementary Table 2). Note that, because these ANOVAs involved Type III sums of squares, each main effect and interaction was evaluated after the variance accounted for by all other main effects and interactions was removed from the model.

Despite inclusion of gender and educational level, the pattern of age and treatment effects remained the same: The main effect of Treatment was still significant for both the Grand Index and the Aggregate Ratings; The main effect of Age Cohort remained nonsignificant for the Grand Index and significant for the Aggregate Ratings; The interaction between Treatment and Age Cohort remained nonsignificant for both the Grand Index and Aggregate Ratings. (Compare Supplementary Table 1 with Table 2a and Supplementary Table 2 with Table 2b.) Moreover, no significant effect on CCT was found for either gender (Treatment x Gender) or educational level (Treatment x Education). The only significant effect of either demographic factor was that of gender on change score for the Aggregate Ratings (Gender, F > M).

Supplementary Table 1. ANOVA results showing the effects of Age Cohort, Treatment, Gender, and Educational Level on change (post - pre) in the NCPT Grand Index.

Source	Type III Sum of Squares	df	Mean Square	F	p
Intercept	28186	1	28186	292.9266	< 2.2e-16 ***
Age Cohort	12	1	12	0.1246	0.72409
Treatment	5848	1	5848	60.7772	7.93e-15 ***
Gender	82	1	82	0.8513	0.35623
Educational Level	199	3	66.3	0.6908	0.55755
Age x Treatment	53	1	53	0.5521	0.45751
Age x Gender	13	1	13	0.1334	0.71490
Age x Education	80	3	26.7	0.2760	0.84279
Treatment x Gender	281	1	281	2.9161	0.08777
Treatment x Education	224	3	74.7	0.7757	0.50741
Gender x Education	16	3	5.3	0.0566	0.98231

Age x Treatment x Gender	0	1	0	0.0006	0.98114
Age x Treatment x Education	163	3	54.3	0.5658	0.63757
Age x Gender x Education	106	3	35.3	0.3671	0.77673
Treatment x Gender x Education	386	3	128.7	1.3375	0.26028
Age x Treatment x Gender x Education	405	3	135	1.4031	0.23988
Error	421555	4381	96.2		

***$p < 0.001$.

Supplementary Table 2. ANOVA results showing the effects of Age Cohort, Treatment, Gender, and Educational Level on change (post - pre) in the Aggregate Survey Rating.

Source	Type III Sum of Squares	df	Mean Square	F	p
Intercept	391.59	1	391.59	1133.2911	< 2.2e-16 ***
Age Cohort	3.39	1	3.39	9.8132	0.001744 **
Treatment	9.64	1	9.64	27.8919	1.345e-07 ***
Gender	3.72	1	3.72	10.7602	0.001045 **
Educational Level	1.54	3	0.51	1.4870	0.215969
Age x Treatment	0.12	1	0.12	0.3508	0.553676
Age x Gender	0.69	1	0.69	1.9869	0.158739
Age x Education	0.33	3	0.11	0.3172	0.812925
Treatment x Gender	0.05	1	0.05	0.1589	0.690219
Treatment x Education	0.88	3	0.29	0.8478	0.467564
Gender x Education	0.27	3	0.09	0.2606	0.853845
Age x Treatment x Gender	0.11	1	0.11	0.3258	0.568146
Age x Treatment x Education	0.62	3	0.21	0.5983	0.616109
Age x Gender x Education	0.72	3	0.24	0.6980	0.553194
Treatment x Gender x Education	1.77	3	0.59	1.7063	0.163456
Age x Treatment x Gender x Education	1.16	3	0.39	1.1172	0.340659
Error	1507.58	4363	0.35		

***p< 0.001. **p< 0.01.
