# Supplementary material for: Computerized Cognitive Training by Healthy Older and Younger Adults: Age Comparisons of Overall Efficacy and Selective Effects on Cognition
Source: Front Neurol. 2021 Jan 8;11:564317. doi: 10.3389/fneur.2020.564317 (PMC7832391; doi:10.3389/fneur.2020.564317)
Supplement: Supplementary file 3 [file Data_Sheet_3.pdf]

CCT Effects on Three Age Cohorts

To explore the extent to which our findings depended on the use of two age cohorts divided at 50, we performed analyses in which the age factor was comprised of three cohorts: young (18-39), middle aged (40-64), and old (65-80). Information about the size and age composition of each cohort is presented in Supplementary Table 3. The three-cohort age and treatment factors were included in ANOVAs on the Grand Index and Aggregate Survey Rating (Supplementary Table 4), as well as in the corresponding ANCOVAs that controlled for baseline differences between participants (Supplementary Table 5). The same patterns of effects were found for the three-cohort age factor as in the prior analyses involving the two-cohort age factor (Tables 2 and 4 in article). Treatment was significant in the ANOVAs and ANCOVAs for both dependent measures. The effect of Age on change score was nonsignificant in the ANOVA and significant in the ANCOVA for the Grand Index; the reverse was found for the Aggregate Survey Rating. The Age x Treatment interaction was nonsignificant in all four analyses.

Supplementary Table 3. Number and ages of participants in each age cohort.

Age Cohort	N	Mean	SD	Median	Skew	Kurtosis
18 – 39	2706	27.34	5.54	27	0.28	-0.87
40 – 64	1731	51.76	6.90	52	0.00	-1.06
65 – 80	278	69.16	3.77	68	0.98	0.27

Supplementary Table 4. ANOVA results showing the effects of Age Cohort and Treatment on change (post - pre) in the NCPT Grand Index **(A)** and Aggregate Survey Rating **(B)**.

A. Change in NCPT Grand Index

Source	Type III Sum of Squares	df	Mean Square	F	p
Intercept	16994	1	16994	176.7825	<2.2e-16 ***
Age Cohort	55	2	27.5	0.2838	0.7529
Treatment	3372	1	3372	35.0731	3.401e-09 ***
Age x Treatment	229	2	114.5	1.1899	0.3044
Error	452685	4709	96.1319		

B. Change in Aggregate of Survey Ratings

Source	Type III Sum of Squares	df	Mean Square	F	p
Intercept	231.95	1	231.95	670.0385	< 2.2e-16 ***
Age Cohort	3.07	2	1.54	4.4299	0.0120 *
Treatment	4.70	1	4.70	13.5778	0.0002 ***
Age x Treatment	0.30	2	0.15	0.4277	0.6520
Error	1622.18	4686	0.3462		

***p< 0.001. *p< 0.05.

Supplementary Table 5. ANCOVA results showing the effects of Age Cohort and Treatment on baseline-adjusted change (post - pre) in the NCPT Grand Index **(A)** and Aggregate Survey Rating **(B)**.

A. Change in NCPT Grand Index

Source	Type III Sum of Squares	df	Mean Square	F	p
Intercept	57537	1	57537	667.4476	< 2.2e-16 ***
Baseline (covariate)	46836	1	46836	543.3190	< 2.2e-16 ***
Age Cohort	9745	2	4872.5	56.5207	< 2.2e-16 ***
Treatment	2950	1	2950	34.2232	5.246e-09 ***
Age x Treatment	177	2	88.5	1.0267	0.3583
Error	405849	4708	86.2041		

B. Change in Aggregate of Survey Ratings

Source	Type III Sum of Squares	df	Mean Square	F	p
Intercept	561.25	1	561.25	2167.2503	< 2.2e-16 ***
Baseline (covariate)	408.92	1	408.92	1579.0348	< 2.2e-16 ***
Age Cohort	1.51	2	0.76	2.9200	0.05403
Treatment	4.68	1	4.68	18.0902	2.148e-05 ***
Age x Treatment	0.62	2	0.31	1.1882	0.30485
Error	1213.26	4685	0.2590		

***p< 0.001.
