# Supplementary material for: Computerized Cognitive Training by Healthy Older and Younger Adults: Age Comparisons of Overall Efficacy and Selective Effects on Cognition
Source: Front Neurol. 2021 Jan 8;11:564317. doi: 10.3389/fneur.2020.564317 (PMC7832391; doi:10.3389/fneur.2020.564317)
Supplement: Supplementary file 5 [file Data_Sheet_5.pdf]

Description of Variables in the Data Set and Analyses

The data set on which the current study is based is contained in Section 4 of the Supplementary Materials. The data set is in csv format and comprises the values of 78 variables (columns) for each of 4715 participants (rows). The variables are listed and described in the table below. Following the table is a list of the ANOVAs and ANCOVAs performed in the study, along with the variables involved in each.

Supplementary Table 6. Names, values, and descriptions of the variables in the study data set.

Column Name	Values	Description
participant_id	integers	unique identifier for each participant
gender	{'m'=male, 'f'=female, 'NA'=not available}	gender
education	{'hs grad or less', 'some college', 'bachelors degree', 'advanced degree', 'NA'=not available}	educational attainment
age_round	integers	age, rounded to the nearest integer
age_bucket	{'o'=older, 'y'=younger}	age group for primary analyses; 'o' is 50 and above
three_age_bins	{'Y'=young, 'M'=middle-aged, 'O'=old}	age group for supplementary analysis; 'Y' is < 40 years old; 'M' is 40-64; 'O' is 65 and older
group	{'crosswords','lumosity'}	training group
four_groups	{'crosswords.o','crosswords.y','lumosity.o' ,'lumosity.y'}	age_bucket appended to group for convenience
active_days	integers	number of training days during the study
est_hours	floating point numbers	estimated total number of hours trained
AR_raw_pre	integers	raw score (number of correct responses) for the Arithmetic Reasoning assessment taken before training
AR_norm_pre	floating point numbers	normalized score for the Arithmetic Reasoning assessment taken before training
TTS_raw_pre	floating point numbers	raw score (negative threshold presentation time) for the Two Target Search assessment taken before training
TTS_norm_pre	floating point numbers	normalized score for the Two Target Search assessment taken before training
GNG_raw_pre	integers	raw score (negative time) for the Go/No-Go assessment taken before training
GNG_norm_pre	floating point numbers	normalized score for the Go/No-Go assessment taken before training
GR_raw_pre	integers	raw score (net number of correct responses) for the Grammatical Reasoning assessment taken before training
GR_norm_pre	floating point numbers	normalized score for the Grammatical Reasoning assessment taken before training
MS_raw_pre	integers	raw score (span) for the Memory Span assessment taken before training
MS_norm_pre	floating point numbers	normalized score for the Memory Span assessment taken before training
PM_raw_pre	integers	raw score (number correct) for the Progressive Matrices assessment taken before training
PM_norm_pre	floating point numbers	normalized score for the Progressive Matrices assessment taken before training
RMS_raw_pre	integers	raw score (span) for the Reverse Memory Span assessment taken before training
RMS_norm_pre	floating point numbers	normalized score for the Reverse Memory Span assessment taken before training
GI_pre	floating point numbers	Grand Index score for the battery of assessments taken before training
AR_raw_post	integers	raw score (number of correct responses) for the Arithmetic Reasoning assessment taken after training

AR_norm_post	floating point numbers	normalized score for the Arithmetic Reasoning assessment taken after training
TTS_raw_post	floating point numbers	raw score (negative threshold presentation time) for the Two Target Search assessment taken after training
TTS_norm_post	floating point numbers	normalized score for the Two Target Search assessment taken after training
GNG_raw_post	integers	raw score (negative time) for the Go/No-Go assessment taken after training
GNG_norm_post	floating point numbers	normalized score for the Go/No-Go assessment taken after training
GR_raw_post	integers	raw score (net number of correct responses) for the Grammatical Reasoning assessment taken after training
GR_norm_post	floating point numbers	normalized score for the Grammatical Reasoning assessment taken after training
MS_raw_post	integers	raw score (span) for the Memory Span assessment taken after training
MS_norm_post	floating point numbers	normalized score for the Memory Span assessment taken after training
PM_raw_post	integers	raw score (number correct) for the Progressive Matrices assessment taken after training
PM_norm_post	floating point numbers	normalized score for the Progressive Matrices assessment taken after training
RMS_raw_post	integers	raw score (span) for the Reverse Memory Span assessment taken after training
RMS_norm_post	floating point numbers	normalized score for the Reverse Memory Span assessment taken after training
GI_post	floating point numbers	Grand Index score for the battery of assessments taken after training
AR_d	floating point numbers	difference in normalized score (post minus pre) on the Arithmetic Reasoning assessment
TTS_d	floating point numbers	difference in normalized score (post minus pre) on the Two Target Search assessment
GNG_d	floating point numbers	difference in normalized score (post minus pre) on the Go/No-Go assessment
GR_d	floating point numbers	difference in normalized score (post minus pre) on the Grammatical Reasoning assessment
MS_d	floating point numbers	difference in normalized score (post minus pre) on the Memory Span assessment
PM_d	floating point numbers	difference in normalized score (post minus pre) on the Progressive Matrices assessment
RMS_d	floating point numbers	difference in normalized score (post minus pre) on the Reverse Memory Span assessment
GI_d	floating point numbers	difference in normalized score (post minus pre) on the NCPT Grand Index
lost_track_details_pre	{1,2,3,4,5,'NA'=not available}	response to a survey item (lost track of details) before training
misplaced_items_pre	{1,2,3,4,5,'NA'=not available}	response to a survey item (misplaced items) before training
lost_concentration_pre	{1,2,3,4,5,'NA'=not available}	response to a survey item (lost concentration) before training
remembered_names_pre	{1,2,3,4,5,'NA'=not available}	response to a survey item (remembered names) before training
felt_creative_pre	{1,2,3,4,5,'NA'=not available}	response to a survey item (felt creative) before training
good_concentration_pre	{1,2,3,4,5,'NA'=not available}	response to a survey item (good concentration) before training
felt_anxious_pre	{1,2,3,4,5,'NA'=not available}	response to a survey item (felt anxious) before training
in_bad_mood_pre	{1,2,3,4,5,'NA'=not available}	response to a survey item (bad mood) before training
felt_sad_pre	{1,2,3,4,5,'NA'=not available}	response to a survey item (felt sad) before training
survey_ave_pre	floating point numbers	average of survey responses before training
lost_track_details_post	{1,2,3,4,5,'NA'=not available}	response to a survey item (lost track of details) after training
misplaced_items_post	{1,2,3,4,5,'NA'=not available}	response to a survey item (misplaced items) after training
lost_concentration_post	{1,2,3,4,5,'NA'=not available}	response to a survey item (lost concentration) after training
remembered_names_post	{1,2,3,4,5,'NA'=not available}	response to a survey item (remembered names) after training
felt_creative_post	{1,2,3,4,5,'NA'=not available}	response to a survey item (felt creative) after training
good_concentration_post	{1,2,3,4,5,'NA'=not available}	response to a survey item (good concentration) after training
felt_anxious_post	{1,2,3,4,5,'NA'=not available}	response to a survey item (felt anxious) after training

`in_bad_mood_post`	{1,2,3,4,5,'NA'=not available}	response to a survey item (bad mood) after training
`felt_sad_post`	{1,2,3,4,5,'NA'=not available}	response to a survey item (felt sad) after training
`survey_ave_post`	floating point numbers	average of survey responses after training
`lost_track_details_d`	{-4,-3,-2,-1,0,1,2,3,4,'NA'=not available}	difference in survey response (post minus pre) for an item (lost track of details)
`misplaced_items_d`	{-4,-3,-2,-1,0,1,2,3,4,'NA'=not available}	difference in survey response (post minus pre) for an item (misplaced items)
`lost_concentration_d`	{-4,-3,-2,-1,0,1,2,3,4,'NA'=not available}	difference in survey response (post minus pre) for an item (lost concentration)
`remembered_names_d`	{-4,-3,-2,-1,0,1,2,3,4,'NA'=not available}	difference in survey response (post minus pre) for an item (remembered names)
`felt_creative_d`	{-4,-3,-2,-1,0,1,2,3,4,'NA'=not available}	difference in survey response (post minus pre) for an item (felt creative)
`good_concentration_d`	{-4,-3,-2,-1,0,1,2,3,4,'NA'=not available}	difference in survey response (post minus pre) for an item (good concentration)
`felt_anxious_d`	{-4,-3,-2,-1,0,1,2,3,4,'NA'=not available}	difference in survey response (post minus pre) for an item (felt anxious)
`in_bad_mood_d`	{-4,-3,-2,-1,0,1,2,3,4,'NA'=not available}	difference in survey response (post minus pre) for an item (bad mood)
`felt_sad_d`	{-4,-3,-2,-1,0,1,2,3,4,'NA'=not available}	difference in survey response (post minus pre) for an item (felt sad)
`survey_ave_d`	floating point numbers	difference in survey averages (post minus pre)

Listed below are the 8 ANOVAs and 6 ANCOVAs reported in the study, along with the variables involved in each. All analyses involved Type 3 sums of squares and were performed using version 4.0.0 of the R statistical program. Those listed in groups A and C were performed on data in wide format using the Anova function in the car package. Those in group B were performed on data in long format using the ezANOVA function in the ez package.

A. Primary analyses: Independent variables = group, age_bucket

1) ANOVA (Table 2a) : Dependent variable = GI_d

2) ANOVA (Table 2b): Independent variable = survey_ave_d

Control for Demographics: Additional Independent variables = gender, education

3) ANOVA (Supplementary Table 1) Dependent variable = GI_d

4) ANOVA (Supplementary Table 2) Dependent variable = survey_ave_d

Control for Compliance: Covariate = active_days

5) ANCOVA (text, Results section) Dependent variable = GI_d

6) ANCOVA (text, Results section) Dependent variable = survey_ave_d

Control for Baseline performance

7) ANCOVA (Table 4a): Dependent variable = GI_d, Covariate = GI_pre

8) ANCOVA (Table 4b): Dependent variable = survey_ave_d, Covariate = survey_ave_pre

B. Secondary analyses: Independent (between-subject) variables = group, age_bucket

9) ANOVA (Table 5): Repeated (within-subject) measures = change on NCPT subtests (AR_d, TTS_d, GNG_d, GR_d, MS_d, PM_d, RMS_d)

10) ANOVA (Table 6): Repeated (within-subject) measures = change on survey items (lost_track_details_d, misplaced_items_d, lost_concentration_d, remembered_names_d, felt_creative_d, good_concentration_d, felt_anxious_d, in_bad_mood_d, felt_sad_d)

C. Analyses with 3-cohort age factor: Independent variables = group, three_age_bins

11) ANOVA (Supplementary Table 4a) : Dependent variable = GI_d

12) ANOVA (Supplementary Table 4b): Independent variable = survey_ave_d

13) ANCOVA (Supplementary Table 5a): Dependent variable = GI_d, Covariate = GI_pre

14) ANCOVA (Supplementary Table 5b): Dependent variable = survey_ave_d, Covariate = survey_ave_pre
